# Osimertinib and Bevacizumab Cotreatment for Untreated EGFR-Mutated NSCLC With Malignant Pleural or Pericardial Effusion (SPIRAL II): A Single-Arm, Open-Label, Phase 2 Clinical Trial

**DOI:** 10.1016/j.jtocrr.2022.100424

**Published:** 2022-10-15

**Authors:** Makoto Hibino, Osamu Hiranuma, Yoshizumi Takemura, Yuki Katayama, Yusuke Chihara, Taishi Harada, Kohei Fujita, Toshiyuki Kita, Nobuyo Tamiya, Takeshi Tsuda, Shinsuke Shiotsu, Yukihiro Tamura, Takashi Aoyama, Yoichi Nakamura, Masaaki Terashima, Yoshie Morimoto, Kazuhiro Nagata, Kenichi Yoshimura, Junji Uchino, Koichi Takayama

**Affiliations:** a Department of Respiratory Medicine, Shonan Fujisawa Tokushukai Hospital, Fujisawa, Japan; b Department of Pulmonary Medicine, Otsu City Hospital, Otsu, Japan; c Department of Pulmonary Medicine, Graduate School of Medical Science, Kyoto Prefectural University of Medicine, Kyoto, Japan; d Department of Respiratory Medicine, Uji Tokushukai Medical Center, Uji, Japan; e Medical Oncology, Fukuchiyama City Hospital, Fukuchiyama, Japan; f Division of Respiratory Medicine, Center for Respiratory Diseases, National Hospital Organization Kyoto Medical Center, Fushimi, Japan; g Department of Respiratory Medicine, National Hospital Organization Kanazawa Medical Center, Kanazawa, Japan; h Department of Respitaroy Medicine, Rakuwakai Otowa Hospital, Kyoto, Japan; i Department of Respiratory Medicine, Toyama Prefectural Central Hospital, Toyama, Japan; j Department of Respiratory Medicine, Japanese Red Cross Kyoto Daiichi Hospital, Kyoto, Japan; k Internal Medicine, Oosumi Kanoya Hospital, Kanoya, Japan; l Department of Respiratory Medicine, Fukuoka University Hospital, Fukuoka, Japan; m Division of Thoracic Oncology, Tochigi Cancer Center, Utsunomiya, Japan; n Department of Medical Oncology, Izumi City General Hospital, Osaka, Japan; o Department of Pulmonary Medicine, Kyoto Kuramaguchi Medical Center, Kyoto, Japan; p Respiratory Center, Koseikai Takeda Hospital, Kyoto, Japan; q Medical Center for Translational and Clinical Research, Hiroshima University, Hiroshima, Japan

**Keywords:** Angiogenesis inhibitor, Bevacizumab, EGFR tyrosine kinase inhibitors, Osimertinib, Vascular endothelial growth factor

## Abstract

**Introduction:**

First-line treatment of *EGFR*-mutated NSCLC with erlotinib plus antiangiogenic inhibitor exhibits promising results. However, the efficacy of this combination has not been fully investigated. Therefore, we evaluated the efficacy and safety of osimertinib plus bevacizumab in patients with *EGFR*-mutated NSCLC complicated with malignant pleural or pericardial effusion (MPE) for whom combination therapy may be particularly effective.

**Methods:**

This single-arm, open-label, phase 2 study aimed to investigate the clinical benefits of the bevacizumab (15 mg/kg) and osimertinib (80 mg) combination in the first-line setting for advanced EGFR-mutated NSCLC with MPE. The primary end point of this study was 1-year progression-free survival (PFS). The secondary end points were objective response rate, PFS, overall survival, drainage-free survival without the need for thoracic or pericardial drainage, and safety.

**Results:**

Between January 2019 and August 2020, a total of 31 patients with *EGFR*-mutated NSCLC were enrolled from Japan in the study. The median PFS was 8.5 months (95% confidence interval [CI]: 5.3–11.3), the 1-year PFS was 32.1% (80% CI: 21.4–43.3), and the objective response rate was 74.2% (95% CI: 56.8–86.3). The median overall survival was not reached. The median drainage-free survival was 18.4 months (95% CI: 10.3–not estimable). Anorexia was the most common grade 3 or higher adverse event (four patients, 12.9%), followed by fatigue and dyspnea (three patients, 9.7%). No treatment-related deaths were recorded.

**Conclusions:**

Osimertinib and bevacizumab combination in patients with advanced *EGFR*-mutated NSCLC with MPE were safe but did not effectively increase PFS when compared with the inferred value from previous literature.

## Introduction

*EGFR* mutations are the most common driver gene mutations in lung cancer among Asian populations and directly affect treatment choice.^1^, ^2^, ^3^, ^4^, ^5^ EGFR tyrosine kinase inhibitors (TKIs) targeting these genes represent key drugs for *EGFR*-mutated advanced NSCLC.^6^, ^7^, ^8^, ^9^ First- (gefitinib and erlotinib), second- (afatinib and dacomitinib), and third-generation (osimertinib) EGFR TKIs are options for first-line treatment.^10^ Among them, single-agent osimertinib has become the primary choice for first-line treatment of *EGFR*-mutated advanced NSCLC on the basis of the results of a randomized phase 3 trial in which prolonged progression-free survival (PFS) and overall survival (OS) were achieved with osimertinib treatment compared with that observed with gefitinib or erlotinib treatment (median PFS, 18.9 versus 10.2 mo; hazard ratio [HR] = 0.46 [95% confidence interval (CI): 0.37–0.57]).^11^ However, because its inhibitory effects are insufficient to achieve complete remission and its use eventually leads to resistance and relapse, a strategy to improve prognosis with first-line treatment is necessary.^12^^,^^13^ A potential treatment option that may address this problem is the combination of an EGFR TKI with human monoclonal antibodies against vascular endothelial growth factor (VEGF); for example, erlotinib and bevacizumab or ramucirumab combination has been found to be an effective first-line regimen with prolonged PFS.^10^^,^^14^, ^15^, ^16^, ^17^

Interestingly, two studies that reported the efficacy of erlotinib and bevacizumab conducted exploratory subanalyses of patients with and without pleural or pericardial effusions, factors that predict poor efficacy of first- and second-generation EGFR TKIs.^17^^,^^18^

In the JO25567 (A randomized phase 2 study comparing erlotinib and bevacizumab combination with erlotinib alone in NSCLC patients harboing EGFR mutaion) study, the median PFS was 9.7 months longer for patients with pleural or pericardial effusion treated with erlotinib plus bevacizumab than for patients treated with erlotinib alone (15.4 mo versus 5.7 mo; HR = 0.45 [95% CI: 0.25–0.82]).^18^ In patients without pleural or pericardial effusion, the median PFS was 5.3 months longer in the erlotinib and bevacizumab combination group than in the erlotinib-alone group (16.4 mo versus 11.1 mo; HR = 0.62 [95% CI: 0.37–1.04]).^18^ In the NEJ026 (Erlotinib plus bevacizumab versus erlotinib alone in patients with EGFR-positive advanced non-squamous non-small-cell lung cancer) study, the median PFS was 4.3 months longer for patients with pleural effusions treated with erlotinib plus bevacizumab than for those treated with erlotinib alone (16.9 mo versus 12.6 mo).^17^ In patients without pleural effusion, the median PFS was 2.4 months longer in the erlotinib plus bevacizumab combination group than in the erlotinib alone group (16.6 mo versus 14.2 mo).^17^ These results suggest that the combination of erlotinib and bevacizumab prolongs PFS, especially in the subgroup of patients with pleural or pericardial effusion.

An important mediator of pleural effusion in patients with lung cancer is VEGF-A, which promotes neovascularization, increases vascular permeability,^19^ and may be associated with resistance to EGFR TKIs.^20^^,^^21^ Furthermore, EGFR expression is up-regulated in the pleural endothelial cells of patients with lung cancer having malignant pleural effusions, and VEGF-A is expressed in exosomes in malignant pleural effusions, increasing proliferation, angiogenesis, and vascular permeability in pleura. Therefore, the synergistic blockade of EGFR and the VEGF receptor may be particularly effective in patients with malignant pleural effusions.^22^

Thus, combination therapy with an EGFR TKI and VEGF inhibitor is theoretically effective, particularly considering that its efficacy has been revealed in clinical trials of first-generation EGFR TKIs, and it is already used in clinical practice.^10^^,^^14^, ^15^, ^16^, ^17^ Promising preliminary investigations of a regimen combining osimertinib—a third-generation EGFR TKI that exhibits the longest PFS as a single agent—and bevacizumab have been conducted.^23^ The safety of osimertinib and bevacizumab as a combined first-line treatment was reported in a phase 1/2 study, but its efficacy has not been fully investigated.^24^ Particularly, the antitumor effect of this treatment on *EGFR*-mutated NSCLC with pleural or pericardial effusion, a condition associated with reduced efficacy of EGFR TKI monotherapy, is unknown. Therefore, we prospectively evaluated the efficacy and safety of osimertinib and bevacizumab cotreatment in patients with *EGFR*-mutated advanced NSCLC with pleural or pericardial effusion who had not previously received systemic chemotherapy for advanced disease.

## Materials and Methods

### Study Design

This single-arm, prospective, open-label, multi-institutional, phase 2 trial (SPIRAL II) evaluated the efficacy and safety of osimertinib and bevacizumab co-treatment in patients with *EGFR*-mutated NSCLC (excluding squamous cell carcinoma) and malignant pleural or pericardial effusion. During the safety confirmation phase, six patients were enrolled, and the study was set to be terminated when safety was not confirmed as per the following criteria: (1) dose-limiting toxicity was confirmed in two or more patients (dose-limiting toxicity was considered grade [G]≥3 nonhematologic toxicity [excluding transient electrolyte abnormalities, hyperglycemia, proteinuria, and hypertension], G4 hematologic toxicity, G4 hypertension, and pneumonitis); and (2) assessments by the independent efficacy and safety evaluation committee. The primary end point was the 1-year PFS rate, as assessed by investigators. The secondary end points were the objective response rate (ORR), PFS, OS, survival not requiring pleural or pericardial drainage (drainage-free survival [DFS]), and safety, as assessed by grading treatment-related toxic effects. This study followed the CONSORT reporting guidelines.^25^

### Study Participants, Eligibility, and Exclusion Criteria

Patients were enrolled between January 2019 and August 2020 and followed up until August 2021, 1 year after the last patient was registered. Eligible patients were determined on the basis of specific inclusion criteria. First, NSCLC must be histologically or cytologically documented, excluding squamous cell carcinoma, categorized as either untreated stage IV (according to the eighth edition of the American Joint Committee on Cancer staging criteria for lung cancer) or recurrent disease after curative treatment. In the case of previous therapy, the following periods must have elapsed since the previous treatment: (1) 4 weeks after the last dose of postoperative adjuvant chemotherapy; (2) 12 weeks after the last dose of definitive thoracic radiation therapy; (3) 2 weeks after the last palliative radiation treatment; (4) 2 weeks after pleural or pericardial drainage therapy; or (5) 4 weeks after other surgeries or procedures. Second, there is a presence of concurrent malignant pleural or pericardial effusion (imaging and clinical evidence of malignancy were considered sufficient even without malignant cytology). Third, illness is caused by sensitizing *EGFR* mutations (including patients with compound *EGFR* mutations). Fourth, the patient should have the ability to receive drugs orally. Fifth, there is a presence of one or more measurable lesions according to the Response Evaluation Criteria in Solid Tumors version 1.1.^26^ Sixth, the patient has an Eastern Cooperative Oncology Group Performance Status of 0 to 2.^27^ Seventh, the patient should have the ability to be hospitalized or placed under equivalent management for at least 2 weeks to undergo the study procedures. Eighth, the patient must be aged 20 years or above at the time of providing informed consent. Ninth, the patient must exhibit normal major organ function (bone marrow, liver, kidney, etc.) and satisfied the following criteria in a test conducted within 2 weeks before registration: (1) neutrophil count greater than or equal to 1500/mm^3^; (2) platelet count greater than or equal to 100,000/mm^3^; (3) hemoglobin level of greater than or equal to 9.0 g/dL; (4) serum aspartate aminotransferase level of less than or equal to 100 IU/L; (5) alanine aminotransferase level of less than or equal to 100 IU/L; (6) serum T-bilirubin level of less than or equal to 1.5 mg/dL; (7) serum creatinine level of less than or equal to 2.0 mg/dL; (8) proteinuria of less than or equal to 1+; and (9) peripheral capillary oxygen saturation (ambient air) greater than or equal to 90%. Tenth, the patient is expected to survive for at least 3 months. And finally, the patient provided written informed consent of their own free will.

Exclusion criteria included the following: (1) previous pleurodesis; (2) interstitial lung disorders such as idiopathic pulmonary fibrosis, interstitial pneumonia, pneumoconiosis, active radiation pneumonia, and drug-induced pneumonia; (3) hemoptysis (expectoration of fresh blood ≥2.5 mL at a time) or current episode or history of bloody sputum that was persistent and lasting more than 1 week, requiring continuous oral hemostatic medication or intravenous hemostatic medication; (4) lung cavity lesions or tumor invasion to a major blood vessel; (5) infectious diseases requiring intravenous antimicrobial or antifungal treatment; (6) corneal ulceration; (7) at risk for any of the following: (a) mean corrected QT (QTc) using the Fredericia method (QTcF) greater than 470 msec, (b) clinically important abnormalities on an electrocardiogram, and (c) any factors that increase the risk of QTc prolongation or arrhythmia, including electrolyte abnormalities (serum potassium <3.6 mmol/L, serum magnesium <1.8 mg/dL, serum calcium <8.8 mg/dL), heart failure, congenital QT prolongation syndrome, family history of QT prolongation syndrome, family history of unidentified sudden death in a first-degree relative younger than 40 years, and use of medications known to prolong the QT interval and induce torsades de pointes; (8) actual or possible pregnancy, lactation, or unwillingness to use contraception; (9) symptomatic brain metastasis; (10) active multiple cancer; (11) poorly controlled diabetes; (12) clinically important complications (such as uncontrolled heart disease, severe arrhythmia requiring medication, persistent watery diarrhea, etc.); (13) severe or uncontrollable systemic disease as determined by the investigator (uncontrollable hypertension, active hemorrhagic diathesis; hepatitis B, hepatitis C, human immunodeficiency virus, or other active infections that would preclude participation in the study or prevent compliance with the protocol); (14) gastrointestinal diseases or refractory nausea and vomiting that may affect the absorption of osimertinib; (15) active wounds; and (16) any characteristics judged inappropriate by the investigators.

Patients with asymptomatic brain metastases with or without previous local treatment were allowed to participate.

Prohibited concomitant medications and therapies included the following: (1) chemotherapy, immunotherapy, hormonal therapy, biological response modifier therapy, radiation therapy, surgery, and thermotherapy for cancer; (2) investigational or experimental antitumor drugs; and (3) drugs known to have strong inducing effects on CYP3A4.

### Study Treatment and Assessment Procedure

All patients were administered 80 mg of osimertinib orally daily and 15 mg/kg of bevacizumab intravenously once every 21 days. Treatments were continued until disease progression, unacceptable toxicity (gastrointestinal perforation, corneal ulcer, interstitial lung disease [ILD] [any grade], pulmonary hemorrhage [G≥2], other hemorrhages, venous thrombosis [G≥3], any nonhematologic toxicity [G≥4], serious arrhythmia, or symptomatic QTcF prolongation), withdrawal of consent, investigator decision to discontinue treatment, or death. Dose interruption of osimertinib and bevacizumab up to 21 and 42 days, respectively, and a single dose reduction of osimertinib to 40 mg/d were permitted. Once a dose had been reduced, it was not re-escalated in subsequent cycles. Dose reduction of bevacizumab was not allowed; the initial dose was used when dosing was resumed. Dose interruption of osimertinib occurred when patients experienced any adverse event (AE) [G≥3], including asymptomatic QTcF prolongation (>500 msec, or a >60 msec increase from baseline). Resumption with dose reduction was allowed when the QTc improved to less than 480 msec (G1) or returned to baseline. When other AEs improved (G≤2), resumption was allowed at the same dose or a single-dose reduction to 40 mg/d. Dose interruption of bevacizumab occurred when patients exhibited any grade of hemoptysis or open wounds, proteinuria (G≥2), or hypertension (G≥3, unless blood pressure could be controlled with antihypertensive medication). Continuation of osimertinib alone was acceptable even when bevacizumab was interrupted. However, when osimertinib administration was interrupted, bevacizumab was also interrupted.

Tumor assessments using computed tomography of the chest and abdomen were performed during screening and every 6 weeks, thereafter for the first 6 months of treatment, and then every 8 weeks until confirmed objective disease progression or death. Brain magnetic resonance imaging was conducted during screening and thereafter when necessary, such as when disease progression was suspected. ORR was defined as the proportion of patients who achieved a complete or partial response as their best overall response.

Routine clinical and laboratory assessments were conducted during screening and throughout the study. Hematology, biochemistry, coagulation, urinalysis, chest radiography, electrocardiography, vital sign monitoring, and physical examinations were performed every 21 days, and echocardiography was conducted during screening and after 42 days of treatment.

AEs were recorded from the beginning of drug administration throughout the treatment period until 30 days after the last dose. AEs were graded using the Common Terminology Criteria for Adverse Events, version 5.0.^28^ When the same AE occurred more than once in the same patient, the largest grade was used.

### Ethical Considerations

This study was conducted in accordance with the principles of the Declaration of Helsinki. Procedures were reviewed and approved by the Clinical Research Network Fukuoka–Certified Review Board (approval number CRB7180004) and the institutional review boards or ethics committees of the participating facilities. Written informed consent was obtained from all patients before any screening or inclusion procedures. This study was registered with the University Hospital Medical Information Network Clinical Trials Registry on July 4, 2017 (identification: UMIN000028071) and with the Japan Registry for Clinical Trials on October 17, 2018 (identification: jRCTs071180004).

### Sample Size Calculation

The FLAURA study (A Phase III, Double-blind, Randomised Study to Assess the Safety and Efficacy of AZD9291 Versus a Standard of Care Epidermal Growth Factor Receptor Tyrosine Kinase Inhibitor as First Line Treatment in Patients With Epidermal Growth Factor Receptor Mutation Positive, Locally Advanced or Metastatic Non Small Cell Lung Cancer.) reported a median PFS of 18.9 months for osimertinib monotherapy in patients with untreated *EGFR*-mutated NSCLC.^11^ A pooled analysis of two phase 2 studies revealed that the median PFS for erlotinib monotherapy in untreated *EGFR*-mutated NSCLC patients with and without pleural or pericardial effusion were 8.0 and 15.3 months, respectively.^29^ Moreover, a subset analysis of the JO25567 study revealed that the median PFS in patients with *EGFR*-mutated NSCLC having pleural or pericardial effusion was 15.4 months when bevacizumab was added to erlotinib monotherapy.^18^ We assumed an HR of 1.91 (15.3 of 8.0) for comparisons of patients with and without pleural or pericardial effusion on erlotinib monotherapy and an HR of 0.52 (8 of 15.4) when bevacizumab was added to erlotinib monotherapy in the same patients. Given the lack of previous research on this topic, we assumed that the risks associated with osimertinib monotherapy for pleural or pericardial effusion and the effects of adding bevacizumab to osimertinib to treat patients with pleural or pericardial effusion were the same as those associated with erlotinib; the median PFS for osimertinib alone and in combination with bevacizumab was estimated to be approximately 10 and 19 months, respectively.

On the basis of these assumed PFS values, the threshold and expected 1-year PFS rates were assumed to be 43.5% and 64.8%, respectively, with a significance level of 10% (one-sided) and a power of 80%, making the number of required patients 27. Assuming a possible dropout rate of 10%, the required sample size was calculated as 30 patients.

### Statistical Analysis

The Kaplan-Meier method was used to estimate median PFS, OS, and DFS; 95% CIs were calculated using the Brookmeyer-Crowley method. Two-sided 80% and 95% CIs for 1-year PFS (one-sided significance levels of 10% and 2.5%, respectively) were calculated using the Greenwood method.^30^ When the lower limit of 80% CI is above the threshold of a 1-year PFS rate of 43.5%, the 1-year PFS was considered statistically significantly superior to the threshold value. Furthermore, the 95% CIs for 1-year OS and DFS rates were estimated using the Greenwood method. The ORR value is presented with 95% CIs following the Wilson method. As an exploratory analysis, PFS and OS were analyzed using the *EGFR* mutation subgroup (exon 21 L858R and exon 19 deletions) as described above, and HRs (exon 21 L858R and exon 19 deletions) were estimated using the Cox regression model. Regarding Safety, AEs were summarized using the Common Terminology Criteria for Adverse Events version 5.0 grade. Statistical analyses were performed using the Statistical Analysis System software version 9.4 (SAS Institute, Cary, NC). Continuous variables are summarized using medians and ranges, and categorical variables are summarized using frequencies and percentages.

All efficacy end points were analyzed on the full analysis set (FAS). The primary end point analysis was evaluated on the per-protocol set (PPS) as a sensitivity analysis. The safety analysis population was defined as all patients who were enrolled in the study and received at least one dose of the study drug.

## Results

### Patient Demographics

**Figure 1:**
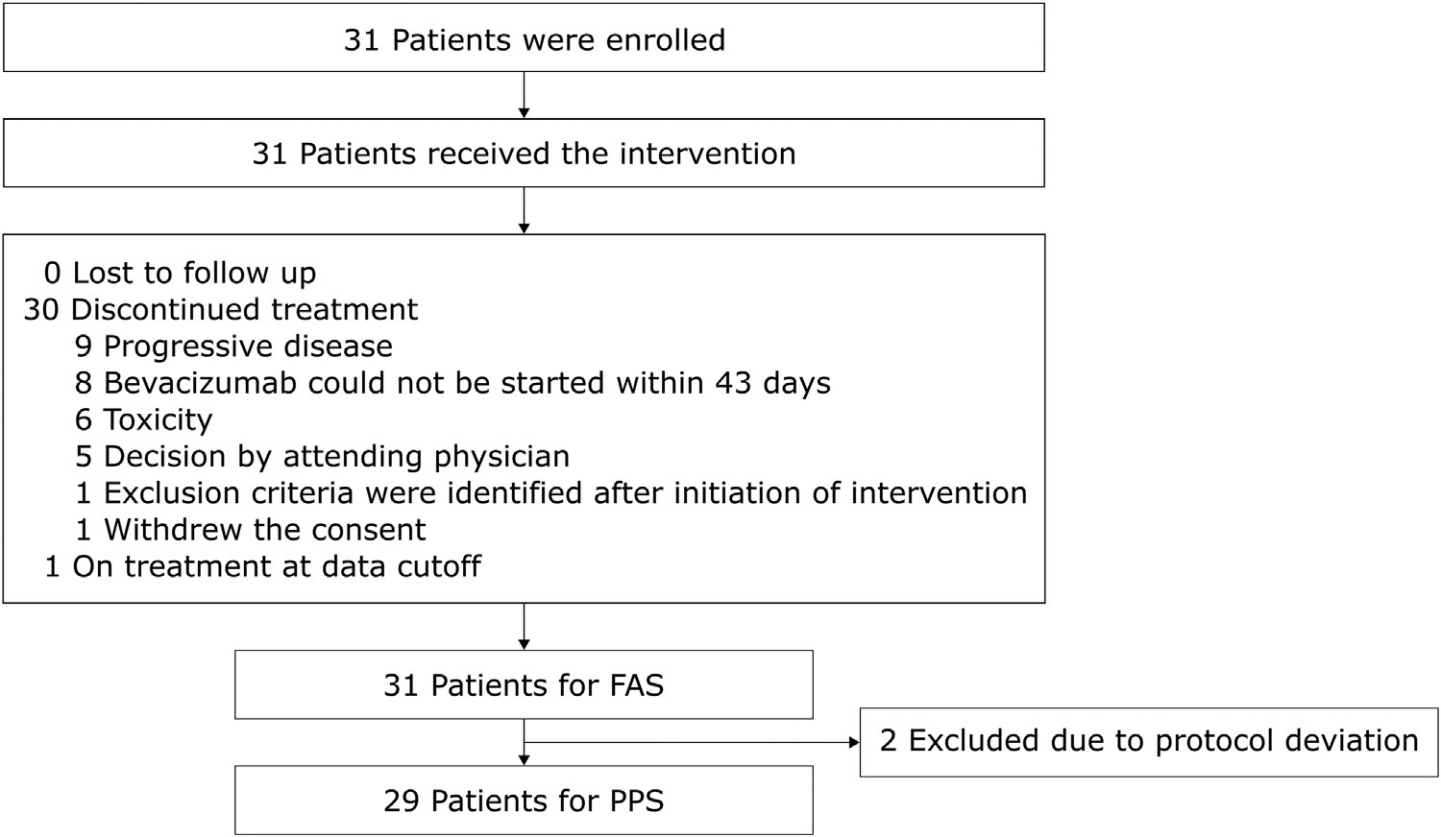
Flow chart illustrating the criteria for patient selection. FAS, full analysis set; PPS, per-protocol set.

A total of 31 patients were enrolled (6 in the safety confirmation phase, plus 25 additional patients) at 15 institutions in Japan from January 2019 to August 2020. Two patients were excluded from the PPS because of protocol deviation—one reported receiving gene therapy at another hospital and the other was enrolled within 2 weeks of the last dose of palliative whole-brain irradiation (Fig. 1).

A total of 20 patients (64.5%) were men, and the median age was 68.0 years (range: 42–85). A total of 30 patients (96.8%) presented with adenocarcinoma, 26 (83.9%) with stage IV disease, and five (16.1%) with postoperative recurrence. A total of 16 patients (51.6%) exhibited exon 19 deletion, 14 (45.2%) were positive for exon 21 L858R, and one (3.2%) was double-positive for exon 21 L858R and exon 20 S768I (Table 1).

**Table 1:** Patient Demographics and Baseline Characteristics

Variables	Number of Patients
	(N = 31)
Sex, n (%)	
Male	20 (64.5)
Female	11 (35.5)
Age (y), median [range]	68.0 (42–85)
Smoking history (former and current), n (%)	16 (51.6)
Histologic subtype of lung cancer, n (%)	
Adenocarcinoma	30 (96.8)
Squamous cell carcinoma	0 (0.0)
Others	1 (3.2)
Clinical stage, n (%)	
Ⅳ	26 (83.9)
Recurrent	5 (16.1)
EGFR mutation, n (%)	31 (100)
Exon 19 deletion	16 (51.6)
Exon 21 L858R	14 (45.2)
Exon 21 L858R and exon 20 S768I	1 (3.2)
ECOG performance status, n (%)	
0	16 (51.6)
1	15 (48.4)
Pleural/Pericardial effusion, n (%)^a^	31 (100)
Pleural effusion	30 (96.8)
Pericardial effusion	3 (9.7)
Metastases, n (%)^a^	26 (83.9)
Lung	9 (29.0)
Ipsilateral	6 (19.4)
Contralateral	6 (19.4)
Pleural dissemination	18 (58.1)
Brain	9 (29.0)
Adrenal gland	3 (9.7)
Bone	14 (45.2)
Liver	4 (12.9)
Extraregional lymph node	4 (12.9)
Previous treatment, n (%)^a^	13 (41.9)
Surgery	6 (19.4)
Postoperative adjuvant chemotherapy	2 (6.5)
Radiotherapy	3 (9.7)
Pleural drainage	2 (6.5)
Pericardial drainage	0 (0.0)
Others	2 (6.5)

ECOG, Eastern Cooperative Oncology Group.

a Multiple choices allowed.

### Efficacy

The median duration of treatment was 4.6 months for osimertinib (range: 0.30–20.5) and 3.4 months for bevacizumab (range: 0.03–19.8). The median follow-up period for PFS and OS was 7.7 months (range: 0.07–21.3) and 15.2 months (range: 0.36–27.3), respectively.

**Figure 2:**
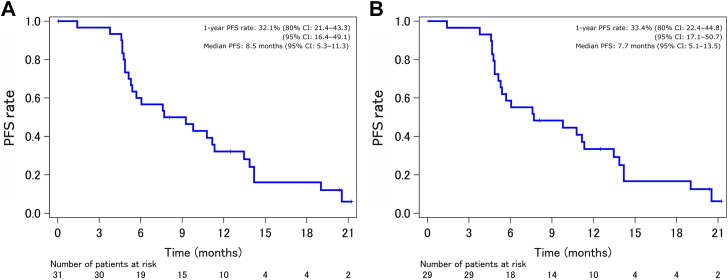
Kaplan-Meier estimate of PFS, full analysis set (*A*, n = 31), and per-protocol set (*B*, n = 29). CI, confidence interval; PFS, progression-free survival.

**Figure 3:**
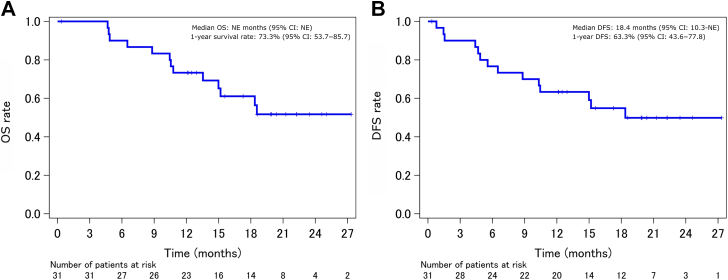
Kaplan-Meier estimates of (*A*) OS and (*B*) DFS, full analysis set (n = 31). CI, confidence interval; DFS, drainage-free survival; NE, not estimable; OS, overall survival.

In the FAS (n = 31), the rate for the primary end point of 1-year PFS was 32.1% (80% CI: 21.4–43.3); the lower limit of this CI was below the threshold of 43.5%. Therefore, this treatment protocol did not surpass the threshold value (Fig. 2*A*). The 1-year PFS rate for PPS (n = 29) was 33.4% (80% CI: 22.4–44.8) (Fig. 2*B*), consistent with that for the FAS. The ORR was 74.2% (95% CI: 56.8–86.3). The overall responses were partial response in 23 patients (74.2%), stable disease in six patients (19.4%), progressive disease in one patient (3.2%), and not assessable in one patient (3.2%); no patients exhibited complete response (0.0%). The median PFS was 8.5 months (95% CI: 5.3–11.3), with events occurring in 26 patients. The estimated median OS was not reached, with events occurring in 13 patients (Fig. 3*A*), and the 1-year OS rate was 73.3% (95% CI: 53.7–85.7). The median DFS was 18.4 months (95% CI: 10.3–not estimable), with events occurring in 14 patients, and the 1-year DFS rate was 63.3% (95% CI: 43.6–77.8) (Fig. 3*B*).

**Figure 4:**
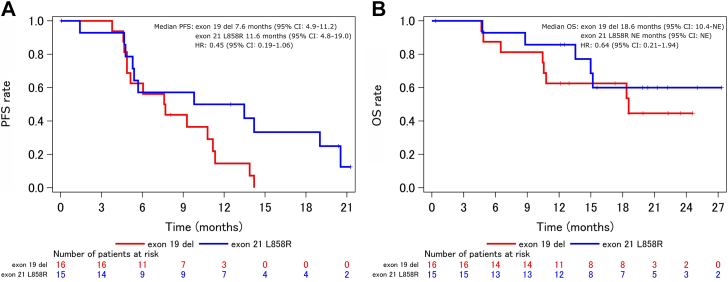
Kaplan-Meier estimates of (*A*) PFS and (*B*) OS. Exploratory subgroup analysis between patients with NSCLC with exon 21 L858R (n = 15) or exon 19 deletion (n = 16). One L858R-positive patient was double-positive for exon 20 S768I. CI, confidence interval; HR, hazard ratio; NE, not estimable; OS, overall survival; PFS, progression-free survival.

**Figure 5:**
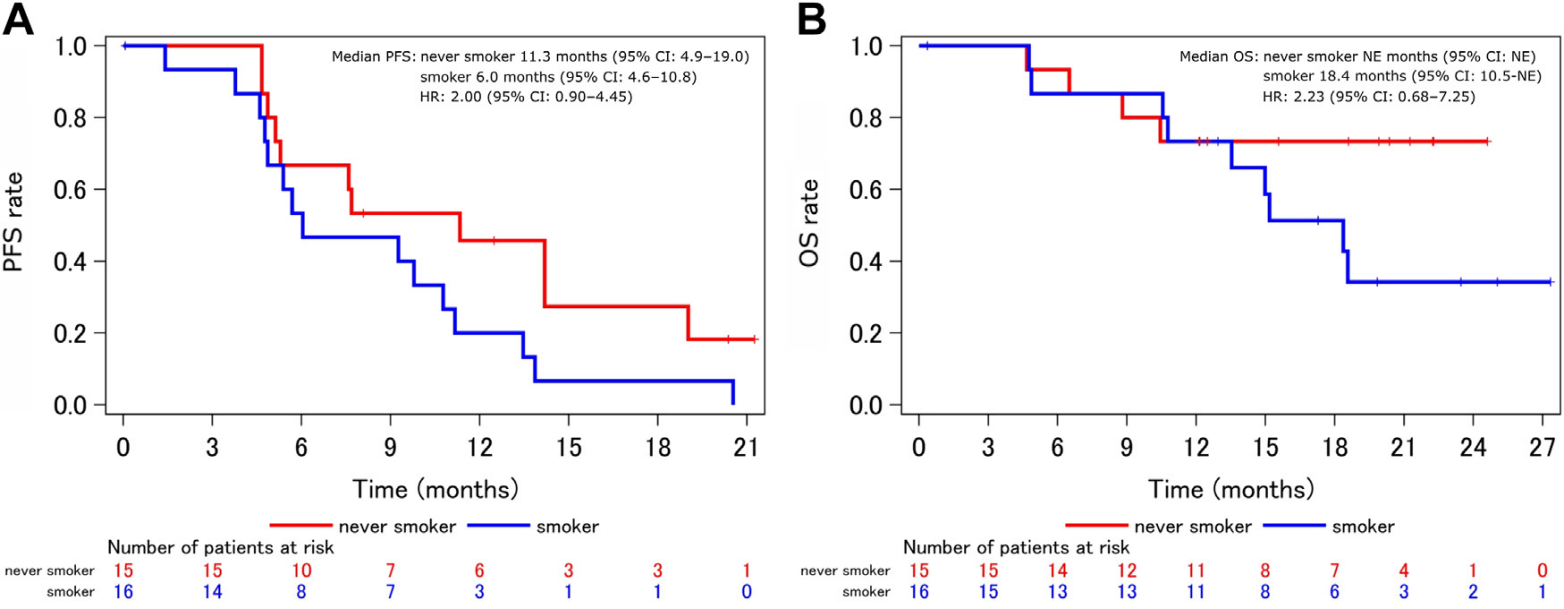
Kaplan-Meier estimates of (*A*) PFS and (*B*) OS. Exploratory subgroup analysis between patients with NSCLC without (n = 15) or with (n = 16) smoking history. CI, confidence interval; HR, hazard ratio; NE, not estimable; OS, overall survival; PFS, progression-free survival.

Additional exploratory subgroup analysis was performed. The median PFS for patients with exon 19 deletion (n = 16) or exon 21 L858R (n = 15) was 7.6 (95% CI: 4.9–11.2) and 11.6 months (95% CI: 4.8–19.0), respectively (HR = 0.45, 95% CI: 0.19–1.06) (Fig. 4*A*). The 1-year PFS rate was 14.6% (95% CI: 2.5–36.8) and 50.0% (95% CI: 22.9–72.2), respectively. The median OS was 18.6 months (95% CI: 10.4–not estimable) and not reached, respectively (HR = 0.64, 95% CI: 0.21–1.94) (Fig. 4*B*). The 1-year OS rate was 62.5% (95% CI: 34.9–81.1) and 85.7% (95% CI: 53.9–96.2), respectively. For patients without (n = 15) and with (n = 16) history of smoking, the median PFS was 11.3 (95% CI: 4.9–19.0) and 6.0 months (95% CI: 4.6–10.8), respectively, (HR = 2.00, 95% CI: 0.90–4.45) (Fig. 5*A*); the 1-year PFS rate was 45.7% (95% CI: 20.1–68.3) and 20.0% (95% CI: 4.9–42.4), respectively; the median OS was not reached and 18.4 months (95% CI: 10.5–not estimable), respectively, (HR = 2.23, 95% CI: 0.68–7.25) (Fig. 5*B*); and the 1-year OS rate was 73.3% (95% CI: 43.6–89.1) and 73.3% (95% CI: 43.6–89.1), respectively.

### Safety

At the data cutoff date, the treatment protocol was ongoing in one patient (3.2%) and had been discontinued in 30 (96.8%); the most common reason for discontinuation was disease progression, which was reported in nine patients (29.0%) (Fig. 1). Of the six patients who met the discontinuation criteria for toxicity, four exhibited interstitial pneumonia (G2, 12.9%), one exhibited hyperkalemia (G4, 3.2%), and one exhibited heart failure (G3, 3.2%). The median duration of bevacizumab therapy in the eight patients who could not start bevacizumab within 43 days was 4.1 (range: 2.4–14.1) months. The most common site of progression in the nine patients who discontinued their regimen owing to disease progression was the lung (six patients); this is followed by the pleura (in the form of pleural effusion [four patients] and pleural dissemination [one patient]), the liver (two patients), and also the pancreas, adrenal gland, bone, muscle, and mediastinal lymph node (one patient each). These included patients who had multiple sites of progression. During the study period, none of the patients discontinued the osimertinib administration and continued bevacizumab monotherapy, 14 patients discontinued bevacizumab before continuing osimertinib monotherapy, whereas 16 patients discontinued both osimertinib and bevacizumab simultaneously.

Treatment-related AEs after the start of treatment in the safety analysis set were generally mild (G1 or G2) (Table 2). The most common G3 or higher AEs were anorexia in four patients (12.9%); fatigue and dyspnea in three (9.7%); and reduced platelet count, increased aspartate aminotransferase, hyponatremia, and heart failure in two (6.5%). No G5 toxicity was detected.

**Table 2:** Treatment-Emergent Adverse Events

Adverse Events	Participants, n (%)						
	n	Grade 0	Grade 1	Grade 2	Grade 3	Grade 4	Any Grade
Fatigue	31	9 (29.0)	15 (48.4)	4 (12.9)	3 (9.7)	—	22 (71.0)
Proteinuria	31	10 (32.3)	6 (19.4)	14 (45.2)	1 (3.2)	—	21 (67.7)
Anemia	31	10 (32.3)	20 (64.5)	1 (3.2)	0 (0.0)	0 (0.0)	21 (67.7)
Anorexia	31	11 (35.5)	9 (29.0)	7 (22.6)	3 (9.7)	1 (3.2)	20 (64.5)
Hyponatremia	31	11 (35.5)	17 (54.8)	1 (3.2)	2 (6.5)	0 (0.0)	20 (64.5)
Platelet count decreased	31	12 (38.7)	14 (45.2)	3 (9.7)	1 (3.2)	1 (3.2)	19 (61.3)
Aspartate aminotransferase increased	31	12 (38.7)	17 (54.8)	0 (0.0)	2 (6.5)	0 (0.0)	19 (61.3)
Alanine aminotransferase increased	31	12 (38.7)	16 (51.6)	2 (6.5)	1 (3.2)	0 (0.0)	19 (61.3)
Dry skin	31	12 (38.7)	18 (58.1)	1 (3.2)	0 (0.0)	—	19 (61.3)
Rash	31	13 (41.9)	14 (45.2)	4 (12.9)	0 (0.0)	0 (0.0)	18 (58.1)
Creatinine increased	31	15 (48.4)	16 (51.6)	0 (0.0)	0 (0.0)	0 (0.0)	16 (51.6)
Paronychia	31	15 (48.4)	11 (35.5)	5 (16.1)	0 (0.0)	—	16 (51.6)
Diarrhea	31	15 (48.4)	13 (41.9)	3 (9.7)	0 (0.0)	0 (0.0)	16 (51.6)
Rash acneiform	31	16 (51.6)	13 (41.9)	2 (6.5)	0 (0.0)	0 (0.0)	15 (48.4)
Dyspnea	31	17 (54.8)	10 (32.3)	1 (3.2)	3 (9.7)	0 (0.0)	14 (45.2)
Cough	31	17 (54.8)	13 (41.9)	1 (3.2)	0 (0.0)	—	14 (45.2)
Hypocalcemia	21	8 (38.1)	11 (52.4)	2 (9.5)	0 (0.0)	0 (0.0)	13 (61.9)
Mucositis, oral	31	19 (61.3)	9 (29.0)	2 (6.5)	1 (3.2)	0 (0.0)	12 (38.7)
Nausea	31	20 (64.5)	6 (19.4)	4 (12.9)	1 (3.2)	—	11 (35.5)
Hypokalemia	31	20 (64.5)	10 (32.3)	0 (0.0)	1 (3.2)	0 (0.0)	11 (35.5)
Alkaline phosphatase increased	30	20 (66.7)	7 (23.3)	2 (6.7)	1 (3.3)	0 (0.0)	10 (33.3)
Neutrophil count decreased	31	21 (67.7)	8 (25.8)	2 (6.5)	0 (0.0)	0 (0.0)	10 (32.3)
Hyperkalemia	31	23 (74.2)	5 (16.1)	2 (6.5)	0 (0.0)	1 (3.2)	8 (25.8)
White blood cell decreased	31	24 (77.4)	0 (0.0)	6 (19.4)	1 (3.2)	0 (0.0)	7 (22.6)
Pruritus	31	24 (77.4)	7 (22.6)	0 (0.0)	0 (0.0)	—	7 (22.6)
Fever	31	26 (83.9)	4 (12.9)	1 (3.2)	0 (0.0)	0 (0.0)	5 (16.1)
Pain	31	26 (83.9)	5 (16.1)	0 (0.0)	0 (0.0)	—	5 (16.1)
Myalgia	31	27 (87.1)	3 (9.7)	0 (0.0)	1 (3.2)	—	4 (12.9)
Pneumonitis	31	27 (87.1)	0 (0.0)	4 (12.9)	0 (0.0)	0 (0.0)	4 (12.9)
Dysgeusia	31	27 (87.1)	2 (6.5)	2 (6.5)	—	—	4 (12.9)
Arthralgia	31	27 (87.1)	4 (12.9)	0 (0.0)	0 (0.0)	—	4 (12.9)
Epistaxis	31	27 (87.1)	4 (12.9)	0 (0.0)	0 (0.0)	0 (0.0)	4 (12.9)
Back pain	31	28 (90.3)	2 (6.5)	0 (0.0)	1 (3.2)	—	3 (9.7)
Blood bilirubin increased	31	28 (90.3)	2 (6.5)	0 (0.0)	1 (3.2)	0 (0.0)	3 (9.7)
Vomiting	31	28 (90.3)	2 (6.5)	1 (3.2)	0 (0.0)	0 (0.0)	3 (9.7)
Constipation	31	28 (90.3)	3 (9.7)	0 (0.0)	0 (0.0)	0 (0.0)	3 (9.7)
Hypermagnesemia	13	11 (84.6)	2 (15.4)	0 (0.0)	0 (0.0)	0 (0.0)	2 (15.4)
Heart failure	31	29 (93.5)	0 (0.0)	0 (0.0)	2 (6.5)	0 (0.0)	2 (6.5)
Malaise	31	29 (93.5)	1 (3.2)	0 (0.0)	1 (3.2)	—	2 (6.5)
Headache	31	29 (93.5)	2 (6.5)	0 (0.0)	0 (0.0)	—	2 (6.5)
Hypernatremia	31	29 (93.5)	2 (6.5)	0 (0.0)	0 (0.0)	0 (0.0)	2 (6.5)
Hypomagnesemia	13	12 (92.3)	1 (7.7)	0 (0.0)	0 (0.0)	0 (0.0)	1 (7.7)
Hypercalcemia	21	20 (95.2)	1 (4.8)	0 (0.0)	0 (0.0)	0 (0.0)	1 (4.8)
Chronic subdural edema	31	30 (96.8)	0 (0.0)	0 (0.0)	0 (0.0)	1 (3.2)	1 (3.2)
Abdominal aortic aneurysm impending rupture	31	30 (96.8)	0 (0.0)	0 (0.0)	1 (3.2)	0 (0.0)	1 (3.2)
Arterial thromboembolism	31	30 (96.8)	0 (0.0)	0 (0.0)	0 (0.0)	1 (3.2)	1 (3.2)
Skin ulceration	31	30 (96.8)	0 (0.0)	0 (0.0)	1 (3.2)	0 (0.0)	1 (3.2)
Esophagitis	31	30 (96.8)	0 (0.0)	0 (0.0)	1 (3.2)	0 (0.0)	1 (3.2)
Gastric ulcer	31	30 (96.8)	0 (0.0)	1 (3.2)	0 (0.0)	0 (0.0)	1 (3.2)
Hematuria	31	30 (96.8)	0 (0.0)	1 (3.2)	0 (0.0)	0 (0.0)	1 (3.2)
Hypertension	31	30 (96.8)	0 (0.0)	1 (3.2)	0 (0.0)	0 (0.0)	1 (3.2)
Papulopustular rash	31	30 (96.8)	0 (0.0)	1 (3.2)	0 (0.0)	0 (0.0)	1 (3.2)
Alopecia	31	30 (96.8)	1 (3.2)	0 (0.0)	—	—	1 (3.2)
Electrocardiogram QT-corrected interval prolonged	31	30 (96.8)	1 (3.2)	0 (0.0)	0 (0.0)	0 (0.0)	1 (3.2)
Peripheral sensory neuropathy	31	30 (96.8)	1 (3.2)	0 (0.0)	0 (0.0)	0 (0.0)	1 (3.2)

## Discussion

In this phase 2 trial, a combination of osimertinib and bevacizumab was administered to patients with previously untreated stage IV or relapsed *EGFR*-mutated NSCLC complicated by malignant pleural or pericardial effusion. Treatment safety was acceptable, but the primary end point was not met on the basis of the 1-year PFS rate.

Although patients in this study were on first-line treatment, osimertinib was initially approved for second-line or later treatment of sensitizing *EGFR*-mutated NSCLC after confirmation of the acquired resistant mutation on exon 20 T790M.^31^, ^32^, ^33^ Therefore, studies to confirm the efficacy of the combination of osimertinib and bevacizumab were conducted in patients with T790M-positive NSCLC who had developed resistance to the preceding first- or second-generation EGFR TKI monotherapy. In the WJOG8715L (A phase I study of osimertinib with bevacizumab and randomized phase II study of osimertinib with or without bevacizumab in EGFR mutated, T790M positive patients who had progressed EGFR-TKIs.) study, a single-arm phase 1B and randomized phase 2 trial, the median PFS was 13.5 and 9.4 months (HR = 1.44, 95% CI: 0.83–2.52) for osimertinib alone and osimertinib plus bevacizumab, respectively, and the study failed to exhibit any benefit of adding bevacizumab to osimertinib.^34^ Similarly, the BOOSTER study (A randomised phase II study of osimertinib and bevacizumab versus osimertinib alone as second-line targeted treatment in advanced NSCLC with confirmed EGFR and acquired T790M mutations), a randomized phase 2 trial, failed to exhibit the efficacy of combination therapy, as no difference in median PFS was seen between combined osimertinib and bevacizumab therapy (15.4 mo; 95% CI: 9.2–18.0 mo) and osimertinib monotherapy (12.3 mo; 95% CI: 6.2–17.2 mo) (HR = 0.96, 95% CI: 0.68–1.37).^35^ These trials reported that this combination was ineffective in patients with disease caused by the *EGFR* T790M mutation that helps develop resistance.^34^^,^^35^ Furthermore, previous exposure to anti-VEGF inhibitors clearly had detrimental effects on second-line treatment with osimertinib plus bevacizumab.^34^ Therefore, this study investigated the efficacy of osimertinib and VEGF inhibitor combination therapy as a first-line treatment.

The phase 2 WJOG9717L trial (Randomized phase II study of osimertinib plus bevacizumab and osimertinib for chemotherapy-naive patients with nonsquamous non-small cell lung cancer harboring EGFR mutations) compared osimertinib plus bevacizumab combination therapy with osimertinib monotherapy in untreated patients having advanced *EGFR*-mutated NSCLC with or without concomitant pleural effusion. A prolonged PFS was not observed as the median PFS in the osimertinib monotherapy arm was 20.2 months (95% CI: 11.79–not estimated) versus 22.1 months (95% CI: 19.81–not estimated) in the combination arm (HR = 0.862, 60% CI: 0.700–1.060, 95% CI: 0.531–1.397, one-sided stratified log-rank *p* = 0.213).^23^

Here, only patients with malignant pleural and pericardial effusion, considered more likely to benefit from bevacizumab and osimertinib combination, were included; but PFS was not prolonged. In our study and the WJOG9717L^23^ trial, PFS was not prolonged, suggesting that bevacizumab does not have an additive antitumor effect when combined with osimertinib, even as a first-line treatment, as it does when used as a second-line or later treatment in T790M-positive patients. The additive effect of the angiogenesis inhibitor was insignificant probably because osimertinib already has high antitumor efficacy. However, more studies are required to determine with certainty why these phase 2 trials did not exhibit an improved efficacy of the combination of osimertinib and an anti-VEGF inhibitor despite the anticipated synergistic antitumor effects.

The PFS observed in our study was shorter than that found in the WJOG9717L^23^ trial, in which patients were treated with osimertinib alone or with osimertinib and bevacizumab, and shorter than that observed in the FLAURA study,^11^ in which patients were treated with osimertinib alone. This result is consistent with the results of the subgroup analysis of the JO25567^18^ and NEJ026^17^ trials of first-generation TKIs with and without pleural effusion. This finding suggested that the presence of pleural or pericardial effusion might indicate a poor prognosis when osimertinib, a third-generation TKI, is used. Although the mechanism underlying the potential of pleural and pericardial effusions as indicators of poor prognosis is unknown, it may involve the function of mast cells in MPEs.^36^ It has been reported that they induce pleural vascular leakage by releasing tryptase AB1 and interleukin-1β and promote fluid accumulation and tumor growth by promoting NF-κB activation.^36^

Some patients may respond poorly to EGFR TKI monotherapy. For example, clinical factors such as malignant pleural or pericardial effusion and leptomeningeal metastasis, and molecular factors such as high programmed death-ligand 1 expression, high tumor mutation burden, and multiple co-occurring genetic alterations (multiple co-mutations) were reported to be associated with reduced effectiveness of EGFR TKI monotherapy.^37^, ^38^, ^39^, ^40^, ^41^, ^42^, ^43^ Besides serosal involvement, another possible explanation for the short PFS observed in this study is the large number of patients who had high levels of programmed death-ligand 1 expression, high tumor mutation burden, or multiple co-mutations, which were not verified in this study. For these populations who previously exhibited a poor response to EGFR TKI monotherapy, new drugs or combination therapies are needed to improve therapeutic efficacy, and these may include combination therapy with an EGFR TKI and VEGF inhibitor. More recently, a phase 2 trial and a retrospective review have reported the potential benefit of osimertinib plus bevacizumab therapy in patients with *EGFR*-mutated advanced NSCLC having leptomeningeal metastasis.^37^^,^^44^ To date, no useful molecular biomarkers have been identified for assessing the efficacy of EGFR TKI treatment combined with a VEGF inhibitor. Regarding clinical biomarkers, Dafni et al.^45^ recently reported that smoking history was associated with prolonged PFS and OS in patients treated with the EGFR TKI and VEGF inhibitor combinatorial therapy. Subgroup analysis of this study revealed a trend toward worse prognosis in the smoking group; however, the design of this study was single-arm, and the effect of smoking history on the effect of combination therapy could not be determined. Therefore, further subcategorization of *EGFR*-mutated NSCLC that can be used to predict responses to combined EGFR TKI and VEGF inhibitor therapy is needed if the development of osimertinib and VEGF inhibitor combination therapy is to continue.

Our safety results revealed that six patients (19.4%) discontinued this treatment owing to AEs. However, no G5 AEs occurred; hence, the drug was considered relatively safe. There was only one case of QT prolongation (G1) and no cases of G3 or higher skin problems that were considered to be associated with osimertinib. G2 ILD occurred in four patients (12.9%), and similar frequencies were observed previously, including the WJOG9717L^23^ and WJOG8715L^34^ trials, in which the incidence of any grade ILD was 18.3 and 12% in the osimertinib arm and 3.3 and 10% in the osimertinib/bevacizumab arm, respectively. G2 hypertension was observed in one patient (3.2%), G1 epistaxis in four (12.9%), and G3 or higher urinary protein in one (3.2%), indicating that the adverse effects associated with bevacizumab are acceptable. This regimen seems acceptably safe when adverse effects are monitored and managed properly.

This study has several limitations. The sample size was small and included only Japanese patients, and the follow-up period was short, potentially affecting the conclusiveness of the results. Second, this was a single-arm study without a comparator group, and the 1-year PFS rate inferred from historical data was used for comparison. Consequently, the efficacy of this treatment cannot be reasonably interpreted. A randomized phase 2 study design comparing osimertinib monotherapy and combined therapy with bevacizumab for patients with lung cancer harboring sensitizing EGFR mutations with malignant pleural or pericardial effusion may eliminate this problem; however, on the basis of the results of this study and the WJOG9717L^23^ trial, such a trial may not be viable.

In conclusion, our study did not meet its primary end point; compared with osimertinib monotherapy, osimertinib and bevacizumab combination treatment failed to increase the 1-year PFS of patients with *EGFR*-mutated advanced NSCLC with malignant pleural or pericardial effusion. The combination of osimertinib and bevacizumab may be evaluated in more selective populations, and combinations of osimertinib with other angiogenesis inhibitors should be further investigated.

## CRediT Authorship Contribution Statement

**Makoto Hibino**: Data curation, Investigation, Methodology, Resources, Writing - Original draft.

**Osamu Hiranuma**: Data curation, Investigation, Resources, Writing - Review & editing.

**Yoshizumi Takemura**, **Yuki Katayama**, **Yusuke Chihara**, **Taishi Harada**, **Kohei Fujita**, **Toshiyuki Kita**, **Nobuyo Tamiya**, **Takeshi Tsuda**, **Shinsuke Shiotsu**, **Yukihiro Tamura**, **Takashi Aoyama**, **Yoichi Nakamura**, **Masaaki Terashima**, **Yoshie Morimoto**, **Kazuhiro Nagata**: Data curation, Investigation, Resources, Writing - Review & editing.

**Kenichi Yoshimura**: Formal analysis.

**Junji Uchino**, **Koichi Takayama**: Conceptualization, Methodology, Writing - Review & editing.

## Data Availability

The data that support the findings of this study are available from the corresponding author on reasonable request.
